# Stressful life events, general cognitive performance, and financial capacity in healthy older adults and Alzheimer’s disease patients

**DOI:** 10.1007/s40211-022-00451-y

**Published:** 2023-01-04

**Authors:** Vaitsa Giannouli, Magda Tsolaki

**Affiliations:** 1 School of Medicine, Aristotle University of Thessaloniki, Thessaloniki, Greece; 2 Department of Psychology, University of Western Macedonia, Florina, Greece

**Keywords:** Assessment, Stress, Financial capacity, Neurocognitive disorders, Old age, Evaluierung, Stress, Finanzielle Kapazität, Neurokognitive Störungen, Hohes Lebensalter

## Abstract

**Background:**

The influence of stressful life events on general cognition and for the first time on financial capacity performance of patients with a diagnosis of Alzheimer’s disease (AD) and in healthy controls (HC) is assessed.

**Methods:**

A total of 268 participants (122 patients and 146 HCs with similar demographics) were examined with a number of neuropsychological tests, including Mini Mental State Examination (MMSE), Geriatric Depression Scale (GDS-15), and Legal Capacity for Property Law Transactions Assessment Scale (LCPLTAS) for measuring financial capacity. The life change unit (LCU) method was also used.

**Results:**

HCs reported more stressful events than AD patients before the onset of the disease as the LCU load was higher for them (51.80 vs. 27.50), but in both groups the level of LCU load was far below 100, which is the threshold suggested for the induction of a psychosomatic disorder. The most frequently reported life event for AD patients was increased family arguments (*n* = 45/122), followed by increase in responsibilities (*n* = 32/122) and financial difficulties (*n* = 29/122), while the HC group reported problems within the family (*n* = 56/146), change in health status (*n* = 32/146), and a death of a beloved family member (*n* = 27/146). Regressions indicate no causal role for recent life events in the etiopathogenesis of AD, but an influence only of MMSE and diagnosis on financial capacity.

**Conclusions:**

Stressful life events do not seem to be important in financial capacity and relevant vulnerability to financial exploitation for either HCs or AD patients; therefore clinicians should not consider them per se as a possible aggravating factor for financial deficits.

## Abstract (de)

**Hintergrund:**

Der Einfluss von belastenden Lebensereignissen auf die generelle kognitive Leistungsfähigkeit und zum ersten Mal auch auf die finanziellen Kompetenz von AD(Alzheimer-Erkrankung)-Patienten und gesunden Kontrollpersonen (HC) wird evaluiert.

**Methoden:**

Insgesamt 268 Probanden (122 AD-Patienten und 146 HC mit ähnlichen demographischen Merkmalen) wurden mit einer Reihe neuropsychologischer Tests untersucht, u. a. Mini Mental State Examination (MMSE), Geriatric Depression Scale (GDS-15) und Legal Capacity for Property Law Transactions Assessment Scale (LCPLTAS) zur Beurteilung von Verständnis und Kompetenzen für finanzielle Angelegenheiten. Außerdem wurde die Methode der „life change unit“ (LCU) angewandt.

**Ergebnisse:**

HC-Patienten gaben mehr belastende Ereignisse an als AD-Patienten vor Ausbruch der Krankheit, die LCU-Belastung war bei ihnen höher (51,80 vs. 27,50), aber in beiden Gruppen lag die LCU-Belastung weit unter 100, dem als für die Auslösung einer psychosomatischen Störung angenommenen Grenzwert. Das von den AD-Patienten am häufigsten berichtete lebensgeschichtliche Ereignis war eine Zunahme familiärer Konflikte (*n* = 45/122), gefolgt von einer Zunahme an Verpflichtungen (*n* = 32/122) und finanziellen Herausforderungen (*n* = 29/122), während die HC-Gruppe von Problemen innerhalb der Familie (*n* = 56/146), Veränderungen des Gesundheitszustands (*n* = 32/146) und dem Tod eines geliebten Familienmitglieds (*n* = 27/146) berichtete. Regressionen weisen darauf hin, dass rezente lebensgeschichtliche Ereignisse keine kausale Rolle in der Ätiopathogenese der AD spielen, sondern nur der MMSE und die Diagnose Einfluss auf die finanzielle Verstehens- und Handlungskompetenz haben.

**Schlussfolgerungen:**

Weder für HC noch für AD-Patienten scheinen belastende Lebensereignisse von Bedeutung zu sein für finanzielle Kompetenzen, auch nicht für die Vulnerabilität gegenüber wirtschaftlicher Ausbeutung. Dies gilt für HC und für AD-Patienten. Daher sollten Kliniker sie nicht per se als einen möglichen erschwerenden Faktor für finanzielle Defizite betrachten.

## Introduction

There is still an open debate on the role of stressful life events as a risk and prognostic factor for dementia [1–4]. More specifically, it has been supported that the experience of only one stressful life event is not associated with dementia incidence, but two or more negative life events do predict higher risk for dementia, but not of Alzheimer’s disease (AD) type [5].

Financial capacity is found to be impaired in many types of neurocognitive disorders and to be further negatively affected by depressive symptomatology, such as in vascular dementia [6], AD [7], and Parkinson’s disease [8], but we still know little about the direct influence of stressful life events (per se) on depressive symptomatology, overall cognition, and financial capacity skills not only of AD patients, but also in healthy controls.

## Methods

The participants were 268 (161 females). Their age ranged from 65 to 98 years (mean [M] = 73.50, standard deviation [SD] = 7.07). Two groups were formed, the first with a diagnosis of AD (*n* = 122; 72 women), and the second healthy controls (HC; *n* = 146; 89 women). Participants were matched regarding their basic demographics, such as age [t(266) = 1.147, *p* = 0.253], gender χ^2^(1) = 0.105, *p* = 0.746, and years of education [t(266) = 0.271, *p* = 0.786]. All participants reported the same socioeconomic status (lower middle-class, based on their annual income and education).

Participants’ diagnosis of AD was made at the Memory and Dementia Outpatient Clinic in G. Papanikolaou General Hospital, Thessaloniki and controls were recruited from the community. Although recruitment took place between June 2013–September 2015 at Thessaloniki, participants came from different parts of Northern Greece. The patients were included consecutively, while the HCs were selected based on their demographics, and in order to match the group of patients. The dropout rate was low (9.15%), given that of the 295 participants, only 27 participants (and/or their caregivers in the case of the AD patients group) refused to be included in the study protocol, mainly due to time restrictions (insufficient time) for the completion of the full examination. This study has been approved by the Ethics Committee of Aristotle University of Thessaloniki (protocol 2.27/3/2013) and was conducted according to the guidelines of the Declaration of Helsinki. Written informed consent was obtained from all patients and their caregivers.

Inclusion criteria were (1) aged ≥ 65 years (in older to define this a homogeneous group of elderly participants), (2) a first (not pre-existing) diagnosis of AD according to the established guidelines from the National Institute of Neurological and Communicative Disorders and Stroke/Alzheimer’s Disease and Related Disorders Association Inc. (NINCDS-ADRDA) and the diagnostic criteria of neurocognitive disorders provided by DSM‑5 (as re-examined after the data collection) at the time of the examination, and (3) Greek native speakers. Exclusion criteria were (1) a history of other neurological or psychiatric illness (e.g., severe mental illness, stroke, epilepsy, sensory impairments not corrected with aids), both ongoing and past, and (2) inexistence of a reliable third source to confirm the existence of stressful events.

General cognition was measured with Mini-Mental State Examination (MMSE), depressive symptomatology was assessed with the 15-item Geriatric Depression Scale and the culturally appropriate cut-off of 6/7 point was applied [9]. None of the participants had a score above this cut-off (M_GDS-15_ = 2.39, SD_GDS-15_ = 3.24). Financial capacity was assessed with the Legal Capacity for Property Law Transactions Assessment Scale (LCPLTAS) [7]. To evaluate stress level, the Social Readjustment Rating Scale (SRRS) or better known as Holmes and Rahe Stress Scale was used. With this scale, each reported event is called a life change unit (LCU) and has a different ‘weight’ for stress. More events mean a higher score and the higher the self-reported score, and the larger the weight of each event, the more likely the patient would become ill. The total score is based on adding the total life change units occurring during the last year, with the following ranges: 0–149 LCU = low stress, 150–299 LCU = moderate stress, and 300 plus LCU = high stress [10]. In this sample, stressful life events ranged from 0–254. In addition to the older person, at least one more person (family member or caregiver) that accompanied them during the neuropsychological assessment, confirmed the existence of the stressful events.

## Results

T‑tests revealed as expected statistically significant differences in MMSE and LCPLTAS, but surprisingly HC reported more stressful events than AD patients (medium effect size, Table 1). Nonparametric bootstrapped estimates of the 95% confidence intervals (CI) for mean difference confirmed the above, through sampling with replacement (1,000 samples of 268 cases) from the original data.

**Table 1:** Demographics, stressful life events, MMSE, GDS-15, and LCPTLAS for the AD group and HC

Variables	Diagnosis	*N*	M	SD	*p*	Cohen’s D effect size
Age	AD	122	74.04	6.24	0.253	–
	HC	146	73.05	7.69		
Education (in years)	AD	122	8.80	4.13	0.786	–
	HC	146	8.95	4.87		
LCU	AD	122	27.50	2.41	0.000	0.55
	HC	145	51.08	57.23		
MMSE	AD	122	24.74	5.21	0.000	3.54
	HC	146	29.41	0.88		
GDS-15	AD	122	2.69	3.73	0.160	–
	HC	146	2.13	2.75		
LCPLTAS	AD	122	69.69	46.86	0.000	4.15
	HC	146	207.56	13.64		

*AD* Alzheimer’s Disease patients, *HC* Healthy Controls, *LCU* Life Change Unit, *MMSE* Mini Mental State Examination, *GDS-15* Geriatric Depression Scale, *LCPLTAS* Legal Capacity for Property Law Transactions Assessment Scale

A linear regression model, “Enter” method (R = 0.970; R^2^ = 0.941), indicated that MMSE (b = 7.724, SE = 0.326, *p* = 0.000) and diagnostic group (b = 20.296, SE = 2.411, *p* = 0.000) predicted the older participants’ LCPLTAS financial capacity scores, but not stressful life events (b = −0.032, SE = 0.027, *p* = 0.243) or depressive symptomatology measured by GDS-15 (b = −0.256, SE = 0.360, *p* = 0.478). Regarding MMSE performance a similar regression (R = 0.876; R^2^ = 0.767) showed that it was predicted only by diagnostic group (b = 6.429, SE = 0.225, *p* = 0.000) and GDS-15 (b = 0.220, SE = 0.067, *p* = 0.001), but not stressful life events (b = −0.003, SE = 0.005, *p* = 0.562).

The single most common (based on frequency) life events for the patient group were an increase in a number of family arguments (*n* = 45/122), followed by increase in responsibilities (*n* = 32/122) and financial difficulties (*n* = 29/122), while for the HC group were problems within the family (*n* = 56/146), change in health status (*n* = 32/146), and a death of a beloved family member (*n* = 27/146).

## Discussion

These preliminary findings support that stressful life events do not trigger the onset of dementia and do not predict general cognition or financial capacity. Of course, both of the groups of this sample are characterized as belonging to the ‘low stress’ category. An interesting new finding that is in contrast to previous literature [8, 11–14] regarding financial capacity is that depressive symptomatology did not negatively influence LCPLTAS scores. This could be due to the low scores of depressive symptomatology as measured by GDS-15 in this sample that can not support a diagnosis of depression as was the case for the population in relevant prior studies [6, 8].

Although there is heterogeneity in the relevant literature regarding the definition and measurement of stress, the above confirm a noticeable financial incapacity in AD patients (lower than 2.5 SDs performance on LCPLTAS compared to controls). Of course, a point that needs careful consideration is that the mean MMSE score of AD patients was 24.74, a value that should receive attention as it implies cognitive impairment, and therefore any self-reports may lack the importance of an objective source. Nevertheless, there was an objective control of the stressful events through the accompanying persons (family members, caregivers) and for the individuals that did not show agreement between their self-reports and their caregivers were excluded in this current sample.

This study has several strengths as the demographic homogeneity of the two groups, but two major limitations are the lack of personality traits examination that may shape perceived stress levels through a longitudinal perspective and the use only of screening instruments for the assessment of psychiatric symptoms. Future research could additionally use diagnostic interviews, while the inclusion of older adults with a diagnosis of mild cognitive impairment (MCI) is also recommended with further examination of these influences over time, while taking into consideration possible delays in first diagnosis of AD based on the lag in years from observation of first symptoms to problem recognition.
